# Spatial Reorganization of Putaminal Dopamine D_2_-Like Receptors in Cranial and Hand Dystonia

**DOI:** 10.1371/journal.pone.0088121

**Published:** 2014-02-10

**Authors:** Kevin J. Black, Abraham Z. Snyder, Jonathan W. Mink, Veeral N. Tolia, Fredy J. Revilla, Stephen M. Moerlein, Joel S. Perlmutter

**Affiliations:** 1 Department of Psychiatry, Washington University School of Medicine, St. Louis, Missouri, United States of America; 2 Department of Neurology, Washington University School of Medicine, St. Louis, Missouri, United States of America; 3 Department of Radiology, Washington University School of Medicine, St. Louis, Missouri, United States of America; 4 Department of Anatomy and Neurobiology, Washington University School of Medicine, St. Louis, Missouri, United States of America; 5 Department of Biochemistry and Molecular Biophysics, Washington University School of Medicine, St. Louis, Missouri, United States of America; 6 Program in Physical Therapy, Washington University School of Medicine, St. Louis, Missouri, United States of America; 7 Department of Neurology, University of Rochester, Rochester, New York, United States of America; 8 Department of Neurobiology and Anatomy, University of Rochester, Rochester, New York, United States of America; 9 Department of Brain and Cognitive Sciences, University of Rochester, Rochester, New York, United States of America; 10 Department of Pediatrics, University of Rochester, Rochester, New York, United States of America; 11 Pediatrix Medical Group, Sunrise, Florida, United States of America; 12 Gardner Family Center for Parkinson’s Disease and Movement Disorders, University of Cincinnati College of Medicine, University of Cincinnati, Cincinnati, Ohio, United States of America; University of Manchester, United Kingdom

## Abstract

The putamen has a somatotopic organization of neurons identified by correspondence of firing rates with selected body part movements, as well as by complex, but organized, differential cortical projections onto putamen. In isolated focal dystonia, whole putaminal binding of dopamine D_2_-like receptor radioligands is quantitatively decreased, but it has not been known whether selected parts of the putamen are differentially affected depending upon the body part affected by dystonia. The radioligand [^18^F]spiperone binds predominantly to D_2_-like receptors in striatum. We hypothesized that the spatial location of [^18^F]spiperone binding within the putamen would differ in patients with dystonia limited to the hand versus the face, and we tested that hypothesis using positron emission tomography and magnetic resonance imaging. To address statistical and methodological concerns, we chose a straightforward but robust image analysis method. An automated algorithm located the peak location of [^18^F]spiperone binding within the striatum, relative to a brain atlas, in each of 14 patients with cranial dystonia and 8 patients with hand dystonia. The mean (left and right) |*x*|, *y*, and *z* coordinates of peak striatal binding for each patient were compared between groups by t test. The location of peak [^18^F]spiperone binding within the putamen differed significantly between groups (cranial dystonia *z*<hand dystonia *z*, *p = *0.016). We conclude that in isolated focal dystonia, dopamine D_2_-like receptors are distributed differently in the putamen depending on the body part manifesting dystonia.

## Introduction

Dystonia is a clinical syndrome of involuntary muscle contractions producing either sustained or intermittent abnormal postures of different body parts [1]. Dystonia may be associated with other neurologic abnormalities or may occur as an isolated manifestation. The isolated dystonias (previously known as primary dystonias) are a diverse group of disorders characterized by involuntary, sustained muscle contractions that may cause twisting and repetitive movements, sustained movements or abnormal postures. These isolated dystonias typically begin in different body parts. Two examples of focal isolated dystonia are cranial dystonia, with sustained involuntary eyelid closure frequently associated with lower facial grimacing, and hand cramp, with excessive co-contractions of agonist and antagonist hand or forearm muscles during specific tasks such as writing.

The pathophysiology of isolated dystonia is only partially understood [2], [3], [4], [5], [6]. However, dopamine D2-like receptor binding in the putamen is abnormally low [2], [7], [8], [9], [10], [11]. Although the neurological manifestation may be focal, it is unclear whether the decrease in D2-like binding is uniform throughout the putamen or whether it is somatotopically related to the body part affected. In other words, D2-like receptors may be more affected in a part of the putamen, corresponding to the focal change in behavior.

We investigated this question by comparing the spatial distribution of [^18^F]spiperone binding in patients with cranial dystonia to the distribution in patients with hand cramp. We hypothesized that if the focal distribution of clinical signs corresponded to a focal abnormality of the putamen, then the spatial distribution of [^18^F]spiperone would differ between the two types of dystonia.

## Methods

### Ethics Statement

The study was approved by the Washington University Human Studies Committee, and all subjects gave written informed consent.

### Patients

A movement disorders specialist examined all subjects and made the diagnosis of isolated cranial dystonia (with predominant involuntary eyelid squeezing and excessive blinking) or isolated hand dystonia based upon typical clinical characteristics [1], [12]. Each person also completed the Edinburgh Handedness Inventory [13], Mini-Mental State Examination [14], and Hamilton Depression Rating Scale [15]. Exclusion criteria included evidence of dementia, depression, dystonia affecting any other part of the body, drug abuse, other neurologic illness or exposure to drugs known to affect dopamine receptors. There were 14 patients with cranial dystonia (1 left-handed, 10 women, median age 54.5, range 46–79), and 8 patients with hand cramp (2 left-handed, 6 women, median age also 54.5, range 25–68). In the hand cramp patients, dystonia affected the writing (dominant) hand predominantly or exclusively. Detailed clinical characteristics of 20 of the 22 subjects were included in a prior report (rows 2–21 in Table 1, ref. [7]). This study adds a 77-year-old right-handed man with blepharospasm for 12 years, taking captopril and theophylline, with botulinum toxin injections 6 months before PET, and a 61-year-old right-handed woman with hand cramp for 12 years, taking estrogens, ergotamine tartrate, caffeine and ibuprofen the day before PET with botulinum toxin injections 3 months before. In a secondary analysis, we examined data from 10 healthy control subjects who were studied contemporaneously using the same methods (1 left-handed, 5 women, median age 63.5, range 24–76).

**Table 1:** Mean location in atlas space of peak [^18^F]spiperone binding, by group (mm).

	|*x*|	*y*	*z*
cranial dystonia	23.9	0.4	2.8
hand cramp	24.5	0.5	4.9
*p*	0.372	0.977	**0.016**
control subjects	24.8	−1.4	4.4

### Magnetic Resonance Image (MRI) Acquisition

Sagittal MPRAGE (TR = 9.7 ms, TE = 4 ms, and flip angle = 12°) images were acquired with a 1.5T Siemens Magnetom scanner [16]. The 3D field of view was 256×256×160 mm with voxel dimensions 1×1×1.25 mm. The main field was shimmed and the transmitter tuned before each study.

### Radioligand

[^18^F]Spiperone was prepared using a microwave-facilitated synthetic pathway [17]. The radiopharmaceutical had ≥95% radiochemical purity and a specific activity ≥2000 Ci/mmol.

### Positron Emission Tomography (PET) Image Acquisition

PET data were acquired with a Siemens ECAT 953b camera in the 2D wobble mode. The spatial characteristics of this instrument have been extensively documented [18], [19]. In the 2D wobble mode it produces 31 2D images with an intrinsic resolution of approximately 5.4 mm (full width half maximum; FWHM) in plane and 4.2 mm axially. Three to 5 mCi of [^18^F]spiperone was injected intravenously, and PET scans began immediately with tracer injection. Scan lengths began at 60 seconds and increased to 10 minutes, for a total of 3 hours. During the scan, patients were at rest with eyes closed in a quiet, darkened room. They were observed frequently and had essentially no dystonic movements during the scan. The images were reconstructed with a ramp filter using attenuation measured by an external ^68^Ge/^68^Ga source.

### Image Processing: Overview

The reconstructed PET images were corrected for inter-scan head movement and registered to a standard atlas [20] using the procedure described below. This procedure yielded, for each subject, an atlas-transformed [^18^F]spiperone PET image weighted to reflect specific binding (“late image”). The late images then were used to examine diagnosis-dependent changes in putaminal [^18^F]spiperone distribution. Although only the late images were analyzed for group differences in [^18^F]spiperone activity, all the acquired PET data were used in computing the PET-MRI image registrations, to maximize the reliability of this critical step ( [21]; see also Supplementary Information in ref. [22]).

### Preliminary PET Image Processing

The decay-corrected, reconstructed PET data acquired during each 3 hour scan were divided into 18 frames, each frame representing 10 minutes of scanning. (The first 3 such frames were created by simple addition of data originally acquired in 2- or 5-minute bins). We called the first 7 ten-minute frames “early” and the last 3 ten-minute frames “late”, leaving 8 “middle” frames. The cutoff times dividing these three groups were chosen to give images on which tracer distribution primarily reflected blood flow (early) or specific binding (late) (see Discussion and refs. [23], [24]). All frames within each group were aligned to the middle frame of that group by rigid body (6 parameter affine) transformations using difference image variance minimization as the objective function [25]. Early, middle and late composite images then were produced by conventional resampling and averaging using 3D linear interpolation.

### PET-MRI Alignments

The early, middle and late composite PET images and the MP-RAGE image were mutually coregistered. Rigid body alignments corresponding to all image pairs within the group were computed using, as the objective function, a variant of the image intensity gradient correlation method of Andersson et al. [26]. On the basis of these alignments, rigid body transforms were computed bringing each of the 3 composite PET images into register with the MP-RAGE. Translational and rotational alignment inconsistency was estimated by comparing the transforms relating each pair of images. The estimated transform inconsistency typically was only ∼0.3 root mean square (rms) mm total for translation and ∼0.3 rms degree total for rotation. In no case did these quantities exceed 0.5 rms mm or 0.5 rms degree.

### Atlas Transformation

Each subject’s MRI was transformed to atlas space by optimizing the linear fit to an atlas target image [20], [25]. Finally, the late PET image was transformed into atlas space by matrix multiplication and trilinear interpolation. The accuracy of this method has been demonstrated [22], [27], [28].

### Peak Search and Statistics

**Figure 1:**
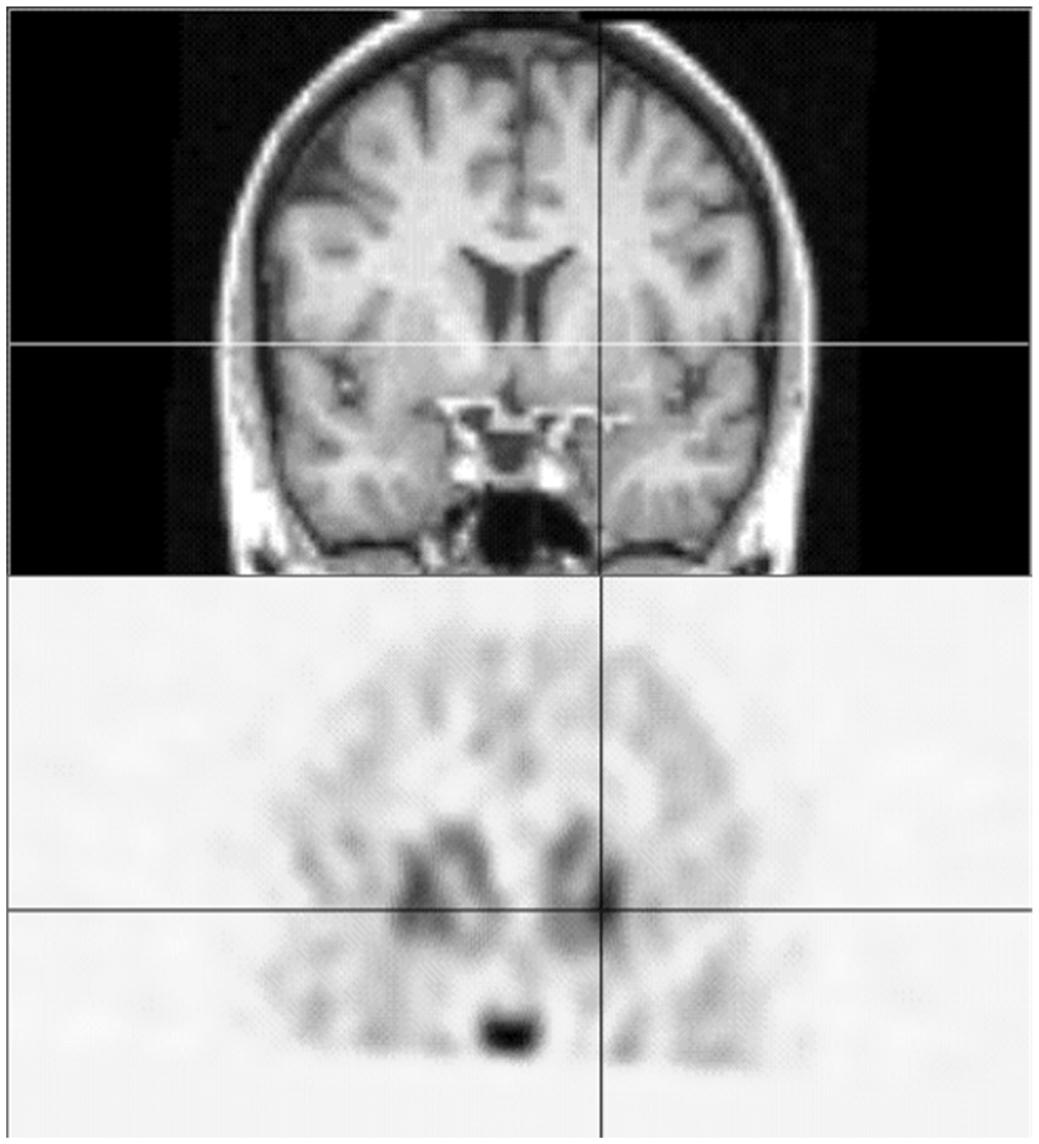
Matched coronal sections from the MP-RAGE (top) and late image (bottom) for one subject. The lines in each image cross at the location of peak putaminal [^18^F]spiperone binding in this subject.

For each subject, the late image was smoothed to 7 mm final FWHM resolution using a Butterworth filter, and then searched with an automated algorithm which reports the center of mass of intensity peaks in the image [29]. Center of mass was computed over a spherical volume of interest with 6 mm radius (see Figure 1 for an example). The coordinates of peak activity in left and right putamen were averaged for each subject using the absolute value of the *x* coordinate. For the cranial dystonia patients no “worse side” can be identified and no left-right difference would be predicted, and many patients with unilateral hand cramp have bilateral physiologic abnormalities [30], [31], [32]. The mean |*x*|, *y*, and *z* coordinates in cranial vs. hand dystonia were compared using unpaired t-tests. We report uncorrected *p* values since the three coordinates are not independent, but note that the conservative Bonferroni correction would accept *p*<0.017 as significant.

## Results

**Figure 2:**
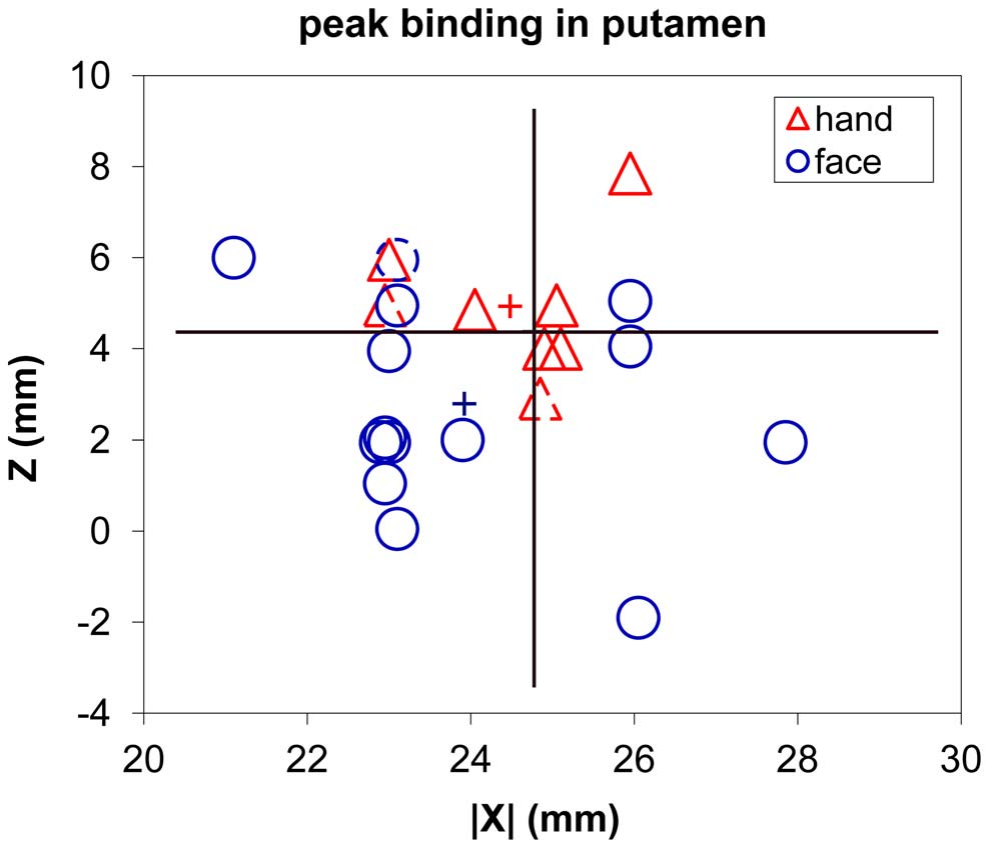
Coronal view of peak [^18^F]spiperone binding in striatum in cranial and hand dystonia. The location of peak [^18^F]spiperone binding in striatum is plotted for each dystonic subject by atlas |*x*| and *z* coordinates according to which part of the body is affected by dystonia. On average, the peak was 2.1 mm more superior in the hand cramp group (red triangles) than in the group with dystonia affecting the face (blue circles). The mean location for each dystonia group is indicated by a plus sign; note that the mean (and median) cranial dystonia peak lies inferior to every hand cramp peak. The three left-handed subjects are indicated by dashed symbols. The large black lines intersect at the mean peak location for the control group.

The peak location of putaminal [^18^F]spiperone binding differed significantly between groups (see Table 1 and Figure 2). Descriptively, the cranial dystonia subjects’ peaks were more medial, anterior and inferior than the hand cramp patients’, but only the *z* dimension difference was statistically significant.

**Figure 3:**
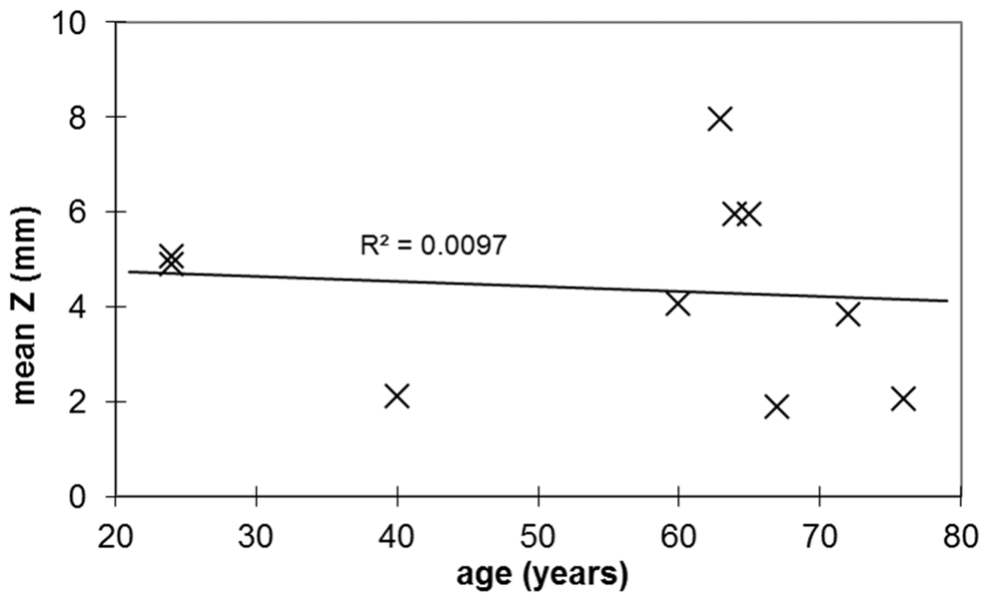
Peak putaminal [^18^F]spiperone binding does not vary significantly with age. The location of peak [^18^F]spiperone binding in striatum (atlas *z* coordinate) is graphed versus age, for a group of normal volunteers. There is no meaningful correlation of this measure with age, and the line that best fits the data has a nearly flat slope.

Since patients in the two dystonia groups could not be perfectly matched by age, we examined whether a difference in mean age was likely to account for the results. This was performed by plotting peak *z* coordinate versus age in a separate group of normal volunteers; images were analyzed exactly as for the dystonia patients. As shown in Figure 3, in this small sample there is no evidence for an age effect (Pearson’s *r* = −0.1). Furthermore, from the best linear fit to this data, the predicted *z* value for the mean age (61.3) of the cranial dystonia patients is 4.3, while the predicted *z* value for the mean age (51.6) of the hand cramp patients is 4.4. The observed difference between the two dystonia groups is 21 times greater than this small difference attributable to age.

## Discussion

The spatial distribution of [^18^F]spiperone binding in the putamen differs in cranial and hand dystonia. This demonstrates differential spatial distribution of receptors within the putamen corresponding to localized behavioral manifestation of dystonia.

We and others have shown that average D_2_-like binding over the whole putamen is 25–30% lower in isolated focal dystonia than in normal controls [7], [8], [9], [10], [11]. (Increased binding of certain D_2_-like ligands in dopa-responsive dystonia is not comparable, as these ligands are displaceable by dopamine, so decreased dopamine synthesis increases their binding even if receptors are normal [33], [34], [35] or decreased [36].) Decreased D_2_-like receptor binding corresponds to numerous other suggestions that dopaminergic dysfunction may be involved in the pathophysiology of focal dystonias [2], [3], [4], [6], [37], [38], [39].

What has not been clear is whether this decrease is homogeneous throughout the putamen. If so, other factors would presumably determine which body part manifests dystonic symptoms. This is consistent with the notion of a “two-hit” animal model of dystonia [40], [41], [42]. Alternatively, different focal dystonias in humans might all feature an average decrease in putaminal D2-like receptor binding, but changes in the distribution of D2-like receptors within the putamen might dictate which body parts were affected. The present study reveals a difference in the spatial distribution of D2-like receptors between patients with dystonia affecting the hand or face, and we speculate that the difference may prove to be somatotopic.

**Figure 4:**
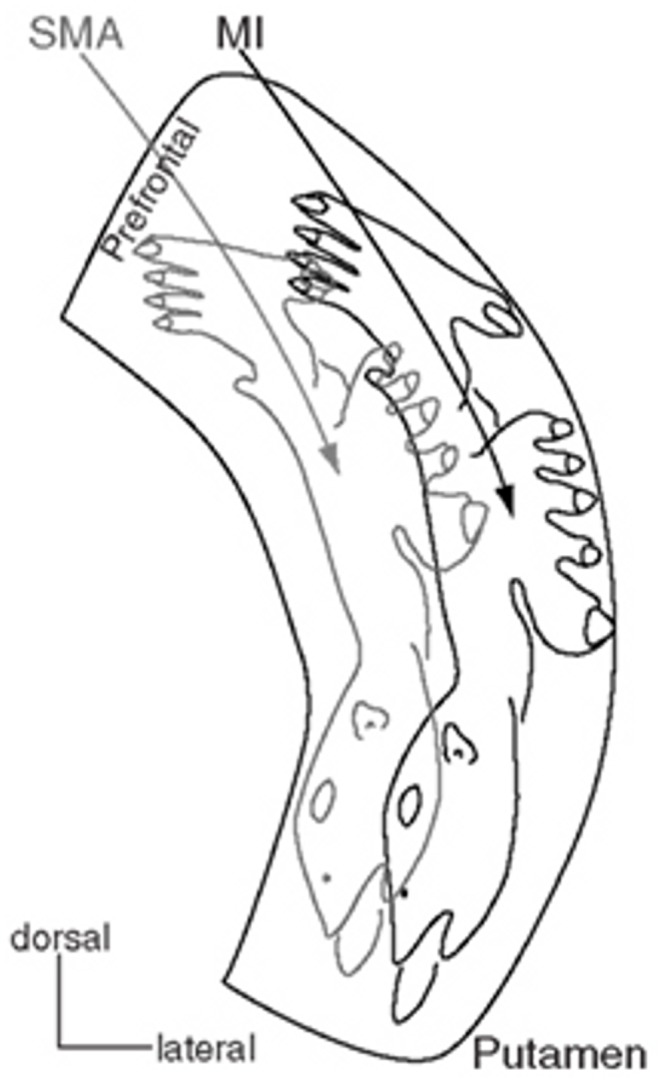
Somatotopy represented on a coronal section of the putamen. Fig. 3A from ref. [43], used by permission.

Several lines of evidence indicate that there is a somatotopic organization in the putamen [43]. Pathway anatomy in monkeys has shown that projections from the face, arm, and leg representations in somatosensory and motor cortex terminate in a topographic pattern that preserves the somatotopic separation of these body areas [44], [45], [46], [47], [48], [49], [50]. The somatotopic arrangement is such that the legs are represented laterally, anteriorly and dorsally in the putamen, the face is represented medially, posteriorly and ventrally, and the arm is represented between these (Figure 4). The somatotopy has been confirmed by studies of neuronal activity related to movement of different body parts and by activation of discrete movements by microstimulation in different areas in putamen [46]. The presence of somatotopy in the human putamen has been shown with functional MRI blood oxygen level dependent (BOLD) activation during self-paced flexion-extension of fingers or toes [51].

The work of Graybiel and colleagues has indicated that the anatomical relationship between somatotopically identified regions of cerebral cortex and putamen is not simple [49]. One area of cortex projects to multiple areas in putamen, and closely related cortical areas (e.g. thumb and fifth finger representations) project to overlapping areas. Thus, there are convergent and divergent projections in the corticostriatal projection [52]. However, projections from non-adjacent body part representations in cortex have little or no overlap in putamen. Thus, despite the complex pattern and multiple representations, face, arm and leg are represented separately. These non-overlapping representations conform to the overall organization described above with leg dorsal, lateral, and anterior to face, and arm in between.

Delmaire and colleagues [53] demonstrated that this normal somatotopic representation in putamen was disrupted in patients with writer’s cramp (task-specific isolated focal dystonia of the hand). In healthy control subjects, repetitive movements of the toes led to BOLD activation in the superior part of the contralateral putamen, lip movements activated the inferior putamen, and finger movement activation in the putamen was between them. By contrast, writer’s cramp patients had no such gradient. As in the present study, the abnormality was limited to the superior-inferior axis.

Several limitations of our work should be addressed. The precise nature of the group difference in distribution of radiotracer binding is not revealed by the methods chosen for this study. Since overall putaminal D2R binding is decreased in focal dystonias, one would like to know whether focal decreases in [^18^F]spiperone binding exist and where they are located within the putamen. Thus a search for a local minimum in [^18^F]spiperone binding in putamen might seem more intuitive. However, numerical searches for local minima in a small region of high signal are likely to identify the putaminal boundary. Another approach would be to compare [^18^F]spiperone binding at each voxel. However, given the available number of subjects and the high image variance, we did not predict adequate power to find a group difference while correcting for multiple comparisons at ∼1000 voxels [54]. Furthermore, the normal spatial distribution of [^18^F]spiperone binding within the putamen is not well characterized at the resolution of PET. Finally, one would like to know whether receptor binding at a given voxel is quantitatively lower or higher than normal. Unfortunately, the counting statistics in [^18^F]spiperone PET images are inadequate to confidently quantify absolute radioligand binding on a pixel-by-pixel basis using validated tracer kinetic analysis techniques. Faced with these difficulties, we chose to characterize the spatial distribution of radiotracer using a straightforward and reliable method, namely location of peak binding in a radiographic image. This method provided adequate statistical power to verify our main hypothesis that putaminal D2-like dopamine receptors are distributed differently in patients with dystonia in different body parts. However, these advantages were bought at a price: this method does not permit us to identify the location in putamen most pertinent to pathophysiology, or to clarify whether the binding at the identified peak is abnormally high or low in either patient group compared to normal. Still, we have shown that there is a difference in D2-like receptor distribution between cranial dystonia and hand cramp.

We use the “late” image alone to make inferences about the location of receptor binding, rather than apply a full tracer analysis to quantify radioligand binding [24], [55]. This is valid in the present context. For valid comparisons of radioligand specific binding between two groups, it is necessary to apply a tracer kinetic model that includes measurements of not only regional radioligand concentration but also regional blood flow, blood volume, time-dependent measures of radioactivity in arterial blood and the accumulation of radiolabeled metabolites. Using these data and parameter estimation methods, it is possible to estimate specific binding of the radioligand binding [7], [23], [24]. However, the present analysis does not require these additional steps, since the peripheral blood measurements are the same for analyzing every part of the putamen, and the small differences that could exist in blood flow and blood volume in different parts of the putamen would not appreciably affect the estimate of radioligand binding [55]. Therefore, identifying the location of peak radioactivity in a single subject is equivalent to identifying the location of peak radioligand binding in that subject, but does not provide an absolute measure of specific binding that would permit quantitative comparisons across subject groups.

Using the whole brain to compute atlas registration improves reliability since there is more data upon which to perform the co-registration, but may be less sensitive to additional changes in receptor distribution which might be detected by a putamen-only atlas. Also, although our results most likely reflect differences in dopamine receptors, only about 70% of [^18^F]spiperone specific binding sites in primate striatum are attributable to D_2_-like dopamine receptors with the remainder composed of serotonin S_2_ receptors [24]. At the time that these studies were performed, [^18^F]spiperone was the only D_2_-like radioligand available to us; more robust receptor measurements may be possible using the D_2_-specific radioligand (*N*-methyl)benperidol [56], [57]. An additional potential limitation in interpreting our results is that the different location of peak binding could reflect either neurochemical or anatomical differences between groups. However, an MRI study in these patients showed no group difference in putamen volume [54], suggesting that the difference is likely to reflect altered receptor distribution rather than anatomical changes. In this study we cannot resolve whether the observed differences in striatal [^18^F]spiperone binding are involved in the production of symptoms or whether they arise in response to the repeated dystonic movements; however, results in a rodent model and in human non-manifesting DYT1 gene carriers are more consistent with the former possibility [10], [58]. Additionally, a recent study suggests that D_3_ rather than D_2_ receptors may be responsible for the abnormal putaminal D_2_-like receptor binding in focal dystonia [57]. Nevertheless, all these issues are tangential to the main finding of a receptor difference in putamen that corresponds to a focal difference in behavior.

In conclusion, patients with dystonia affecting different body parts have a different spatial distribution of [^18^F]spiperone binding in the putamen that may reflect the previously demonstrated somatotopic organization of putamen. This demonstrates that focal behavioral manifestations of disease can correspond to focal neurotransmitter receptor changes in striatum.
